# Effectiveness of a clinical practice change intervention in increasing the provision of nicotine dependence treatment in inpatient psychiatric facilities: an implementation trial

**DOI:** 10.1186/s12888-017-1220-7

**Published:** 2017-02-07

**Authors:** Paula M. Wye, Emily A. Stockings, Jenny A. Bowman, Chris Oldmeadow, John H. Wiggers

**Affiliations:** 1 School of Psychology, University of Newcastle, University Drive, Callaghan, New South Wales 2308 Australia; 2 Hunter Medical Research Institute, Lot 1 Kookaburra Circuit, New Lambton Heights, New South Wales 2305 Australia; 3 Hunter New England Population Health (HNEPH), Longworth Ave, Wallsend, New South Wales 2287 Australia; 4 National Drug and Alcohol Research Centre, University of New South Wales, 22-32 King Street, Randwick, New South Wales 2031 Australia; 5 School of Medicine and Public Health, University of Newcastle, University Drive, Callaghan, New South Wales 2308 Australia

**Keywords:** Tobacco use disorder, Smoking, Psychiatric department, hospital, Psychiatric nursing, Clinical trial, Interrupted time series analysis, Health plan implementation, Health planning guidelines, Clinical practice nursing research, Healthcare systems

## Abstract

**Background:**

Despite clinical practice guidelines recommending the routine provision of nicotine dependence treatment to smokers in inpatient psychiatric facilities, the prevalence of such treatment provision is low. The aim of this study was to examine the effectiveness of a clinical practice change intervention in increasing clinician recorded provision of nicotine dependence treatment to patients in inpatient psychiatric facilities.

**Methods:**

We undertook an interrupted time series analysis of nicotine dependence treatment provision before, during and after a clinical practice change intervention to increase clinician recorded provision of nicotine dependence treatment for all hospital discharges (aged >18 years, *N =* 4175) over a 19 month period in two inpatient adult psychiatric facilities in New South Wales, Australia. The clinical practice change intervention comprised six key strategies: leadership and consensus, enabling systems and procedures, training and education, information and resources, audit and feedback and an on-site practice change support officer. Systematic medical record audit and segmented logistic regression was used to determine differences in proportions for each nicotine dependence treatment outcome measure between the ‘pre’, ‘during’ and ‘post-intervention’ periods.

**Results:**

The prevalence of all five outcome measures increased significantly between the pre and post-intervention periods, including clinician recorded: assessment of patient smoking status (36.43 to 51.95%; adjusted odds ratio [AOR] = 2.39, 99% Confidence Interval [CI]: 1.23 to 4.66); assessment of patient nicotine dependence status (4.74 to 11.04%; AOR = 109.67, 99% CI: 35.35 to 340.22); provision of brief advice to quit (0.85 to 8.81%; AOR = 97.43, 99% CI: 31.03 to 306.30); provision of nicotine replacement therapy (8.06 to 26.25%; AOR = 19.59, 99% CI: 8.17 to 46.94); and provision of nicotine dependence treatment on discharge (8.82 to 13.45%, AOR = 12.36; 99% CI: 6.08 to 25.14).

**Conclusions:**

This is the first study to provide evidence that a clinical practice change intervention may increase clinician recorded provision of nicotine dependence treatment in inpatient psychiatric settings. The intervention offers a mechanism for psychiatric facilities to increase the provision of nicotine dependence treatment in accordance with clinical guidelines.

**Electronic supplementary material:**

The online version of this article (doi:10.1186/s12888-017-1220-7) contains supplementary material, which is available to authorized users.

## Background

Smoking remains a leading global cause of death and disability [1]. While smoking rates and the corresponding burden of premature death and disability have declined in the general population, this has not been observed among persons with a mental disorder [2, 3]. As a consequence, smoking rates are two to three times higher [4], nicotine dependence is more severe [4], quit attempts are less likely to be successful [5, 6], and rates of smoking related morbidity and mortality are substantially higher among persons with such a disorder [7, 8]. Smoking prevalence and its’ associated burden appear to increase with greater severity of mental illness [9], with higher prevalence among psychiatric inpatients [10]. Reduction of the prevalence of smoking in this population is both a recognised public health priority and a clinical priority for mental health services [11, 12].

Despite clinical practice guidelines [13, 14] recommending the provision of nicotine dependence treatment to patients in mental health treatment settings in developed nations such as the United States, United Kingdom and Australia [13–15], the provision of such treatment in psychiatric facilities has been suboptimal, with assessment of nicotine dependence and provision of nicotine replacement therapy (NRT) in inpatient psychiatric facilities occurring rarely (0%–0.5%) [16, 17]. Reported barriers to the provision of such treatment include lack of time and clinician skill and experience, and lack of organisational support, even when comprehensive nicotine dependence treatment guidelines are in place [18].

Systematic review evidence supports the effectiveness of a number of clinical practice change strategies for improving health-related practices across a range of clinical settings, including: engaging local opinion leaders [19], audit and feedback [20], reminders [21], clinical decision support systems [22], training and education [23], the development and dissemination of clinical practice guidelines [24], and dissemination of educational materials [22]. Such strategies found to be effective in increasing the implementation of nicotine dependence treatment specifically, include staff training and education [25], and use of electronic health records [26]. A systematic review and meta-analysis of controlled studies demonstrated that such clinical practice change strategies can increase staff provision of assistance to quit to patients in hospital settings (pooled risk difference: 16.6; 95% confidence interval: 4.9 to 28.3) but no effect was found for staff assessment of smoking status, advice to quit, or the provision or discussion of NRT [27]. The review identified no studies that reported the effectiveness of clinical practice change interventions in increasing the implementation of nicotine dependence treatment in inpatient psychiatric facilities.

To address this evidence gap, a study was undertaken to assess the effectiveness of a multi-modal clinical practice-change intervention in increasing clinician recorded provision of five recommended elements of nicotine dependence treatment: 1) assessment of smoking status; 2) assessment of nicotine dependence; 3) provision of brief advice to quit; 4) provision of NRT; and 5) provision of nicotine dependence treatment at discharge to patients admitted to inpatient psychiatric facilities.

## Methods

### Study design

An interrupted time series study was undertaken. A clinical practice change intervention was implemented for nine months, with outcome data collected for three periods over 19 months: ‘pre-intervention’ (five months from 1st June to 31^st^ October 2009), ‘intervention period’ (nine months from 1^st^ November 2009 to 31^st^ July 2010), and ‘post-intervention’ (five months from 1^st^ August to 31^st^ December 2010).

### Setting

The study was undertaken concurrently in a convenience sample of two of four general locked adult inpatient psychiatric facilities in one health district in New South Wales, Australia. One facility (100 beds) consisted of a discrete psychiatric facility catering for approximately 2000 patient discharges per annum across five units: an emergency/intensive care unit, two general acute units, a dual diagnosis unit, and an older persons unit. The second psychiatric facility (125 beds) catered for approximately 750 patient discharges per annum from a single general acute psychiatric care unit. Both facilities introduced a smoke-free policy in all buildings and grounds three years prior to the study.

### Participants

The study participants consisted of all adult (>18 years) patient discharges, including repeat discharges for individual patients, and for both voluntary and involuntary patients.

### Nicotine dependence treatment

All clinical staff (approximately 170), including registered nurses, nurse managers, allied health professionals, and medical/psychiatric staff received the intervention. Based on both local and international clinical guidelines [13, 14], clinical staff were asked to provide the following five elements of nicotine dependence treatment: 1) assessment of smoking status of all patients on admission; 2) assessment of nicotine dependence; 3) provision of quit advice; 4) provision of NRT (including nicotine patch, lozenge, gum, or inhaler); and 5) provision of any component of nicotine dependence treatment at discharge (including any of brief advice to quit, offer of referral to the Quitline telephone support service, and provision of NRT at discharge). Other forms of pharmacotherapies for nicotine dependence treatment, including varenicline and bupropion were not available to patients through the hospital pharmacy, and nicotine oral and nasal sprays were not approved for use in Australia at the time of the study.

### Clinical practice change intervention

The following strategies, informed by clinical practice change evidence [19–24] and evidence of strategies to improve nicotine dependence treatment in healthcare settings [25–27] were implemented to support the routine provision of nicotine dependence treatment by clinical staff:

- Leadership and consensus
  - Consultation with senior medical, nursing and facility management staff
  - Unit level meetings with managers, and clinical leaders and staff using the principles of motivational interviewing [28];
  - Meetings with consumer advocates, and;
  - Appointment of local (minimum of 1 per unit) clinical champions.
- Enabling systems and procedures
  - Distribution of state nicotine dependence treatment guidelines, the *NSW Health Guide for the Management of Nicotine Dependent Inpatients* [14] (see Additional file 1) to clinical staff, and;
  - Modification of existing admission forms to prompt recording of smoking treatment.
- Training and education
  - o Face-to-face staff group training sessions (*n =* 30) to increase awareness, agreement and adoption of nicotine dependence treatment guidelines;
  - o Problem solving with local champions and clinician groups on ‘as needs’ basis (*n* = 33), and;
  - o Information sessions for patients and carer groups.
- Practice change information and resources
  - o All clinical staff and managers were provided: a treatment flowchart (see Additional file 1); a guide for providing and recording treatment; tailored information regarding smoking and mental health and use of NRT; educational resources for patients; and information regarding support for smoking abstinence or cessation by staff.
- Audit and feedback
  - o Audit and feedback of unit medical records regarding prevalence of nicotine dependence treatment (*n =* 14 face to face information sessions held in total with clinical staff across the six units to provide tailored feedback on the performance of their unit).
- Practice change Support

A practice change support officer (0.5 FTE) was available to support delivery of the above strategies.

### Data collection procedures

All components of the medical record for each patient discharged during the study period were audited post-discharge by trained auditors employed by the research team [16, 29]. Auditors were non-clinical health service staff based in the hospital medical records department, were not specifically linked to or affiliated with the psychiatric facilities and were independent of the study team. Auditors were aware that the audit pertained to tobacco use, but were not privy to the intervention activities.

### Measures

- Patient characteristics

The following patient characteristics were obtained from the medical record: gender, age (determined by date of birth), admission and discharge dates, previous admissions (yes, no), number of discharges during study period), treating unit at discharge, Aboriginal or Torres Strait Islander status (yes, no, unknown), English speaking (yes, no, unknown). Patient diagnosis was classified via ICD-10 codes [30].

b) Nicotine dependence treatment

Based on the *NSW Health Guide for the Management of Nicotine Dependent Inpatients* [14], see Additional file 1) the recorded provision of five elements of nicotine dependence treatment was audited:

1. assessment of smoking status (yes, no);
2. assessment of nicotine dependence (yes, no);
3. provision of brief advice to quit (yes, no);
4. prescription of any form of NRT (including nicotine patch, lozenge, gum, or inhaler) (yes, no), and;
5. provision of nicotine dependence treatment on discharge record (any of: provision of written information/brief advice to quit, offer of referral to Quitline, and provision of NRT post discharge) (yes, no).

### Analysis

Analyses were conducted using SAS version 9.4 software [31]. Admission and discharge dates were used to calculate length of stay. Diagnoses were collapsed into eight most prevalent categories: schizophrenia/psychosis; affective disorders; adjustment/anxiety disorders; substance use disorders; borderline personality disorder; bipolar disorder; dementias, and other mental disorder. For smoking status assessment and provision of brief advice outcomes, audit data from multiple medical record locations were collapsed to form a single measure (recorded/not recorded anywhere).

Descriptive statistics were used to summarise patient characteristics, and prevalence of each of the nicotine dependence treatment measures at each of three time periods (pre-intervention, intervention and post-intervention). Chi-square analyses were undertaken to assess differences in patient socio-demographic and clinical characteristics between patients with one or more admission, and between study periods.

Similar to other studies of nicotine dependence treatment in hospital settings based on medical record audit [32, 33], each outcome measure is reported as a proportion of the total patient sample (both smokers and non-smokers). Smoking prevalence was assumed to be constant during the study period, based on previous research conducted in this facility [16, 34], and elsewhere [2]. The proportions for each outcome were plotted for each fortnight in the study period [35]. Segmented logistic regression analysis of interrupted time series data was used to determine differences in outcome proportions between the pre- and during, during and post-, and pre to post-intervention periods, to plot outcome proportions across the 19-month study period [36]. Month was the unit of time chosen to generate the slopes in the logistic regression analyses, and as such there were five time points (i.e. months) in the pre-intervention period (1st June to 31^st^ October 2009), nine time points in the ‘intervention period’ (1^st^ November 2009 to 31^st^ July 2010), and five time points in the ‘post-intervention’ period (1^st^ August to 31^st^ December 2010). The models adjusted for differences in patient socio-demographic and clinical characteristics (including discharging unit) between study periods. All discharges were treated independently in the analyses. All statistical tests were two-tailed, and the threshold for statistical significance was *p* < 0.01. We used the conservative 1% threshold for statistical significance (and corresponding 99% Confidence Intervals [CIs]) in an attempt to correct for an increased overall type I error rate from the multiple tests (and intervals) that were conducted.

## Results

### Patient characteristics

There were 4,175 discharges (1054 pre-intervention; 2054 during intervention and 1078 post-intervention) during the 19 month study period for 2,898 individual patients. Nearly one third of total discharges (*n =* 1277, 30.6%) were for patients readmitted to the facilities during the study period. There were no differences in socio-demographic or clinical characteristics between patients who had one or multiple admissions.

Across the total study period, patients were mostly male (55%), had an average age of 39.2 years (SD = 15.3) and an average length of stay of 17.0 days (SD = 33.4). The most prevalent primary diagnoses on admission were schizophrenia and related psychosis (25%), adjustment disorders (18%) and unipolar affective disorders (17%). Primary diagnosis on admission, and discharging unit were identified to vary significantly between study periods (*p* < .0001), and these variable were added as confounding variables to the segmented logistic regression models (Table 1).

**Table 1:** Socio-demographic and clinical characteristics of the patient sample at the three audit periods, pre- (*n =* 1054), during (*n =* 2043) and post-intervention (*n =* 1078)

	Time period				
Variable	Pre intervention (*n =* 1054)	Intervention period (*n =* 2043)	Post intervention (*n =* 1078)	Total sample (*n =* 4175)	*P*
Gender					0.8397
Male	573 (54%)	1110 (54%)	597 (55%)	2280(55%)	
First admission to facility?					0.1123
Yes	410 (39%)	841 (41%)	402 (38%)	1653(40%)	
Planned discharge?					0.3193
Yes	1019 (99%)	2004 (99%)	1064 (99%)	4087(99%)	
Discharging unit					<.0001
Emergency and intensive care	93 (9%)	364 (18%)	241 (23%)	698(17%)	
General acute unit 1	235 (23%)	334 (16%)	152 (14%)	721(17%)	
General acute unit 2	226 (22%)	465 (23%)	164 (15%)	855(20%)	
Comorbid mental health and substance use unit	209 (20%)	341 (17%)	207 (19%)	757(18%)	
Rural acute unit	223 (21%)	443 (22%)	243 (23%)	909(22%)	
Older persons unit	52 (5%)	89 (4%)	66 (6%)	207(5%)	
Identified as Aboriginal or Torres Strait Islander?					0.0325
Yes	83 (7.9%)	199 (9.8%)	121 (11%)	403(10%)	
No or unknown	968 (92%)	1840 (90%)	955 (89%)	3763(90%)	
English speaking					0.0232
Yes	1019 (97%)	1994 (98%)	1062 (99%)	4075(98%)	
No or unknown	32 (3.0%)	46 (2.3%)	14 (1.3%)		
Primary diagnosis on admission					<0.0001
Schizophrenia and related psychosis	262 (25%)	524 (26%)	251 (23%)	1037(25%)	
Unipolar affective disorder	204 (19%)	320 (16%)	170 (16%)	694(17%)	
Bipolar disorder	91 (8.7%)	204 (10%)	108 (10%)	403(10%)	
Adjustment disorder	181 (17%)	355 (17%)	201 (19%)	737(18%)	
Borderline personality disorders	104 (10%)	190 (9%)	74 (7%)	368(9%)	
Other mental disorder	40 (4%)	60 (3%)	34 (3%)	134(3%)	
Drug use disorders	118 (11%)	277 (14%)	198 (18%)	593(14%)	
Dementias	13 (1.2%)	12 (0.6%)	5 (0.5%)	30(1%)	
Other medical condition	34 (3%)	93 (5%)	36 (3%)	163(4%)	
Age in years					0.1345
mean (SD)	39.0 (15.5)	38.9 (15.0)	40.0 (15.5)	39.2(15.3)	
Length of stay					0.5144
mean (SD)	16.1 (35.9)	17.6 (34.4)	16.7 (28.4)	17.0 (33.4)	

### Nicotine dependence treatment

The following results were robust to adjustment for potentially confounding variables, and did not differ between patients who had one or multiple admissions. Adjusted results are shown in text, and unadjusted results are provided in Table 1 in the Additional file 1.

1. Assessment of smoking status

**Fig. 1:**
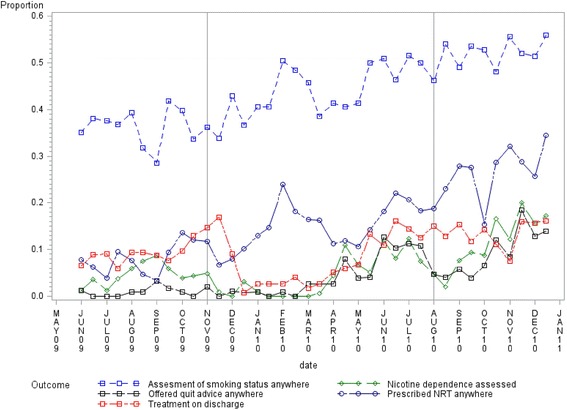
The proportions (yes) of each of the five nicotine dependence treatment outcomes, plotted fortnightly for the total patient sample at pre- (1^st^ June-31^st^ October, 2009, *n =* 1054), during (1^st^ November 2009–31^st^ July 2010, *n =* 2043) and post-intervention (1^st^ August-31^st^ December 2010, *n =* 1078)

The prevalence of recorded assessment of patient smoking status increased significantly between each of the study periods, with an overall significant increase from pre- to post-intervention (15.52% absolute increase, 99% Confidence Interval [CI]: 10.04 to 20.99; Table 2). As shown in Fig. 1 and Table 3, overall, the odds of smoking status being assessed increased significantly from pre-intervention to the end of the post-intervention period (adjusted odds ratio [AOR] = 2.39, 99% CI: 1.23 to 4.66), driven by increases from the pre- to intervention period (Fig. 1, Table 3).

**Table 2:** Prevalence (%), and change in prevalence (%) of clinician recording of the five key nicotine dependence treatment items for the total patient sample within, and between the three audit periods, pre- (*n =* 1054), during (*n =* 2043) and post-intervention (*n =* 1078)

	Prevalence %(yes), *n* (99% CI)			Change in prevalence % (99% CI)		
Outcome	Pre intervention (*n =* 1054)	Intervention period (*n =* 2043)	Post intervention (*n =* 1078)	Pre-intervention vs Intervention	Intervention vs Post-intervention	Pre-intervention vs Post-intervention
Smoking status assessed	36.43, *n =* 384 (32.61, 40.25)	43.47, *n =* 888 (40.64, 46.29)	51.95, *n =* 560 (48.03, 55.87)	7.03 (2.28, 11.78)^**^	8.48 (3.65,13.31)^**^	15.52 (10.04, 20.99)^**^
Nicotine dependence assessed	4.74, *n =* 50 (3.06, 6.43)	4.16, *n =* 85 (3.02, 5.30)	11.04, *n =* 119 (8.58, 13.50)	−0.58 (-2.62, 1.45)	6.88 (4.17, 9.59)^**^	6.30 (3.31, 9.28)^**^
Provided quit advice anywhere in medical record	0.85, *n =* 9 (0.12, 1.58)	4.11, *n =* 84 (2.98, 5.24)	8.81, *n =* 95 (6.59, 11.04)	3.26 (1.91, 4.60)^**^	4.70 (2.21, 7.20)^**^	7.96 (5.62, 10.30)^**^
Prescribed any form of NRT on the medication chart	8.06, *n =* 85 (5.90, 10.23)	14.88, *n =* 304 (12.85, 16.91)	26.25, *n =* 283 (22.80, 29.71)	6.82 (3.85, 9.78)^**^	11.37 (7.37, 15.38)^**^	18.19 (14.12, 22.26)^**^
Treatment for smoking at discharge	8.82, *n =* 93 (6.57, 11.08)	7.78, *n =* 159 (6.26, 9.31)	13.45, *n =*145 (10.77, 16.13)	−1.04 (1.68, -3.76)	5.67 (8.75, 2.59)^**^	4.63 (8.12, 1.13)^**^

*CI* confidence interval

^**^Indicates significant at *p* < .01

**Table 3:** Results of segmented logistic regression showing differences in odds of reporting of the key nicotine dependence treatment items within, and between the pre- (*n =* 1054), during (*n =* 2043) and post-intervention (*n =* 1078) periods

	Slope AOR^a^ within period^b^ (99% CI)			Difference in slope AOR^a^ between periods^c^ (99% CI)			
Outcome	Pre intervention (*n =* 1054)	Intervention period (*n =* 2043)	Post intervention (*n =* 1078)	Pre- intervention vs. intervention	Intervention vs. Post-intervention	Pre-intervention- vs. Post-intervention	End of pre-intervention vs. end of post-intervention^d^
Smoking status assessed	0.99 (0.93, 1.05)	1.02 (0.97, 1.07)	1.02 (0.82, 1.21)	1.03 (1.01, 1.05)^**^	0.98 (0.81, 1.18)	1.01 (0.79, 1.29)	2.39 (1.23 to 4.66)^**^
Nicotine dependence assessed	1.06 (0.93, 1.20)	1.23 (1.11, 1.37)^**^	2.22 (1.63, 3.03)^**^	1.16 (1.10, 1.24)^**^	1.80 (1.33, 2.43)^**^	2.10 (1.42, 3.09)^**^	109.67 (35.35, 340.22)^**^
Provided brief advice to quit	1.07 (0.80, 1.42)	1.23 (1.11, 1.37)^**^	2.22 (1.63, 3.03)^**^	1.23 (1.15, 1.32)^**^	1.73 (1.23, 2.40)^**^	2.11 (1.37, 3.24)^**^	97.43 (31.03, 306.30)^**^
Prescribed NRT	1.05 (0.95, 1.16)	1.10 (1.02, 1.19)^**^	1.32 (1.05, 1.66)^**^	1.05 (1.01, 1.08)^**^	1.19 (0.96, 1.49)	1.25 (0.94, 1.66)	19.59 (8.17, 46.94)^**^
Treatment for smoking on discharge	0.97 (0.90, 1.04)	0.98 (0.92, 1.03)	1.79 (1.46, 2.20)^**^	1.01 (0.98, 1.04)	1.84 (1.51, 2.24)^**^	1.85 (1.43, 2.40)^**^	12.36 (6.08, 25.14)^**^

^**^Indicates significant at *p* < .01

*AOR* Adjusted odds ratio, *CI* Confidence interval, *NRT* Nicotine replacement therapy

^a^Odds ratios were adjusted for potential confounders. Unadjusted odds ratios are presented in the Additional file 1. Adjustment for cofounders did not affect results

^b^Represents the change in recording of treatment within each period (accounting for all months in each period)

^c^Represents the change in recording of treatment between each period (accounting for all months in each period)

^d^Represents the change in recording of treatment from the last data point of the pre-intervention period to the last data point of the post-intervention period

2. Assessment of nicotine dependence

The prevalence of recorded assessment of nicotine dependence was stable in the pre-intervention phase, and increased significantly from intervention to post-intervention, with an overall significant increase from pre- to post-intervention (6.30% absolute increase, 99% CI: 3.31 to 9.28; Table 2). As shown in Fig. 1 and Table 3, overall the odds of a nicotine dependence assessment being recorded increased significantly from pre-intervention to the end of the post-intervention period (AOR = 109.67, 99% CI 35.35, 340.22; Table 3), with increases in odds occurring between each time period (Fig. 1, Table 3).

3. Provision of brief advice to quit

The prevalence of recorded brief advice to quit increased significantly between each of the study periods, with an overall significant increase from pre-to post intervention (7.96% absolute increase, 99% CI: 5.62 to 10.30; Table 2). As shown in Fig. 1 and Table 3, the odds of recorded provision of brief advice to quit increased significantly from pre to post-intervention (AOR = 97.43, 99% CI 31.03, 306.30), with increases in odds occurring between each time period (Table 3).

4. Prescription of NRT

The prevalence of recorded prescription of NRT increased significantly between each of the study periods, with an overall significant increase from pre-to post-intervention (18.19% absolute increase, 99% CI: 14.12 to 22.26; Table 2). As shown in Fig. 1 and Table 3, the odds of recorded prescription of NRT increased significantly from pre- to post-intervention (AOR = 19.59, 99% CI: 8.17 to 46.94), with this being driven by increases from the pre- to intervention period (Table 3).

5. Provision of nicotine dependence treatment at discharge

The prevalence of recorded provision of nicotine dependence treatment on the discharge summary was stable from pre- to during-intervention, but increased significantly from during to post-intervention, with an overall significant increase from pre- to post-intervention (4.63% absolute increase, 99% CI: 1.13 to 8.12). As shown in Fig. 1 and Table 3, the odds of recorded provision of nicotine dependence treatment at discharge increased significantly from pre- to post-intervention (AOR = 12.36, 99% CI: 6.08 to 25.14), with this being driven by increases from the intervention to post-intervention period (Table 3).

Stratified results for the 100-bed (~2000 discharges per annum) and 125-bed facility (~750 discharges per annum) are shown in Tables 2 and 3 of the Additional file 1. The direction and strength of the associations in the two facilities was largely similar to the overall aggregated findings. However, due to the lower number of annual admissions in the 125-bed facility, the number of events was small and power was reduced, resulting in wide confidence intervals around the estimates for this facility (see Additional file 1: Table 3).

## Discussion

This is the first study to examine the effectiveness of a clinical practice change intervention in increasing the recorded provision of nicotine dependence treatment in inpatient psychiatric facilities. The findings suggest that the intervention was effective in increasing clinician-recorded provision of nicotine dependence treatment, with increases identified for all five nicotine dependence treatment measures during the intervention period, and continuing for up to five months post-intervention.

The magnitude of the observed improvement in recording of nicotine dependence treatment in our study are similar to [18], and in some cases greater than [37] those reported in previous studies conducted in general hospital facilities. For example, a four-year interrupted time series study utilising a similar multi-component clinical practice change intervention in 37 general public hospitals in Australia [38] found significant increases in recorded provision of nicotine dependence treatment from pre to post-intervention, including: advice to quit (8.6% increase vs. 8% in our study); and provision of inpatient NRT (16% vs. 18%) [38]. Similarly a controlled study conducted across four hospitals in the same state as the current study identified increases in nicotine dependence treatment that ranged from 9 to 22% [39]. It is particularly encouraging that in the present study, the increases in nicotine dependence treatment occurred in inpatient psychiatric settings—settings known to have higher rates of patient smoking [10], and negative staff attitudes to delivering smoking treatment [40].

The observed rates of recorded provision of the five nicotine dependence treatment variables were stable across the pre-intervention period, and increased in accordance with the timing of the delivery of the clinical practice change intervention. Critically, these increases continued into the post-intervention period, suggesting not only the efficacy of the clinical practice change intervention, but some degree of sustainability of effect. This finding adds to the growing body of evidence that multimodal nicotine dependence treatment interventions show efficacy in increasing nicotine dependence treatment provision in hospital settings both during, and after implementation of the intervention [38]. Further, the observed increases across multiple elements of nicotine dependence treatment are noteworthy, as previous studies have indicated improvements in some but not all forms of treatment assessed [27, 38]. This finding is particularly important, as there is evidence that patient receipt of multiple elements of such treatment increases the likelihood of stopping smoking [13, 41, 42]. It is possible that the lower baseline proportions of patients receiving nicotine dependence treatment in our study (e.g. 0.85% for quit advice) compared to general medical settings (e.g. 5.4% for quit advice in Slattery et al.’s [38] study), paired with the higher rates of smoking and nicotine dependence in these samples relative to the general population [3, 10] meant that there was greater scope for improvement to be made and detected across all nicotine dependence outcomes in the current study. Further, there are inherent differences across studies in intervention length, intensity, follow-up length and outcome measurement that may have differentially influenced the results across these studies, the degree to which is unknown.

Notwithstanding the consistent increases in all outcomes, at post-intervention, an assessment of smoking status occurred for only 52% of patients, with even lower rates for other elements of nicotine dependence treatment. This suggests that additional strategies may be required to ensure that all patients receive nicotine dependence treatment. Previous research has indicated that interventions that are integrated with, and address existing hospital systems and procedures may maximise nicotine dependence treatment delivery [13, 32, 39, 43]. For example, enhancing hospital accountability by including nicotine dependence treatment indicators in hospital accreditation and performance processes has been suggested to increase the provision of such care [44, 45]. Similarly, incorporating nicotine dependence treatment within existing hospital computer systems (such as an electronic medical record system) has been shown to increase smoking care, and may also reduce clinician time burden [32]. The improved, but still suboptimal rates of smoking status assessment and nicotine dependence treatment at post-intervention in this study may have been due to the absence of such strategies in the intervention, and/or the length of the intervention period. Little research has investigated the intervention strategy intensity or duration required for successful guideline implementation and clinical practice change [19, 20]. Similarly, due to the complex, multi-modal and interrelated nature of the intervention components used in the current study, we are unable to comment on which single component—or combinations of components—had the greatest effect, which may be important for implementation in clinical settings where resources may be limited. Future researchers in this area may consider examining the effect of individual intervention components and collecting data regarding intervention implementation costs, in order to advise cost-effectiveness analyses.

This study has multiple strengths, including its’ large sample size, inclusion of a number of patient diagnostic groups, and use of systematically independently collected medical record audit data. However, a number of study characteristics may have impacted on its findings. Firstly, we relied on medical record audit as a measure of clinical staff provision of nicotine dependence treatment. It is possible that clinical staff may have recorded care delivery in the absence of it occurring, and hence overestimated actual delivery of nicotine dependence treatment. However, systematic review evidence suggests that medical record audit is more likely to underestimate clinical staff behaviour [46]. Although the audit staff were non-clinical health service staff who were independent of the research team and were not privy to the intervention activities, they may have become aware of the intervention process via being part of the broader health system. Thus we cannot exclude the possibility that the audit staff may have recorded data more rigorously in the post-intervention period than before. Further, although the hospital nicotine dependence treatment guidelines recommend a clear ‘pathway’ of care (see Additional file 1) where smoking status is assessed, brief advice is provided, NRT is prescribed and patients are monitored until discharge, it is unclear from the audit data to what extent clinical staff followed this pathway in a linear matter. For example, NRT may have been provided before (or in the absence of) provision of brief advice to quit, particularly so for patients who may be well known to clinical staff due to repeat admissions. While it is out of scope here to comment on each individual patient’s nicotine dependence treatment pathway, future studies in this area may aim to examine both global increases care delivery (as demonstrated here), but also improvements in adherence to the care pathway as detailed in clinical practice guidelines.

Secondly, given that the recording of smoking status did not occur systematically for all patients at any time point during the study, the true sample of smokers for whom nicotine dependence treatment should be delivered was unknown. Previous research conducted among patients in the same facility within the same timeframe indicated that smoking rates were approximately 53% [47, 48]. Hence, the rates of nicotine dependence treatment presented here are likely to underestimate the true proportion of smokers receiving nicotine dependence treatment. Nevertheless, assuming that the prevalence of smoking status remained stable across the study period [32, 33], the observed increases in nicotine dependence treatment provision over time are considered to likely reflect an increase in nicotine dependence treatment delivery to patients who are smokers.

Third, given our focus on increasing clinician-recorded nicotine dependence treatment according to the standard hospital care guidelines, we did not collect data on other factors that may have had an impact on the likelihood of clinical staff providing and recording such treatment to patients. This may have included the voluntary or involuntary status of patients, which has shown to be important in the implementation of smoke-free policies in locked psychiatric facilities, given the relative abilities of these patients to leave the grounds to smoke. While we did not collect such data in our study, in Australia in 2014–15 it was estimated that 31.1% of all hospitalisations to specialised psychiatric care services were for patients who had an involuntary admission [49]. These two groups may represent distinct clinical cohorts for whom there may have been a differential effect of the intervention [50]. We also did not collect data regarding patient use of other pharmacological treatments for smoking cessation outside of those provided as part of standard hospital care guidelines, including varenicline and bupropion (which were not available through the hospital pharmacy) or oral or nasal nicotine spray (which was not approved for use in Australia at the time of the study). The use of such other pharmacotherapies outside of those provided as standard hospital care may have influenced the likelihood of clinicians providing routine hospital nicotine dependence treatment to patients.

Further, utilising a controlled trial design was not feasible, and the use of a non-controlled design constrains our ability to directly attribute the observed increases in recording of nicotine dependence treatment to the intervention component. Given the limited resources available, the study facilities were required to be within close geographical proximity to the administering University, and were too dissimilar in size and annual patient admissions in order for one site to act as a comparator to the other. As such, the interrupted time series design was adopted, as it has been demonstrated to be appropriate in evaluating the efficacy of clinical practice change interventions in hospital settings where whole-of-system practice change is the focus of the intervention [38]. Further, this pragmatic study design provides one means of accelerating the research translation process by simultaneously disseminating the intervention strategy whilst conducting the evaluation [51]. Where feasible, future researchers aiming to examine the efficacy of a clinical practice change intervention should consider the inclusion of a comparison condition, such as through the use of a randomised controlled trial design (e.g. [52]) or through hybrid evaluation designs, such multiple baseline (e.g. [53]) or stepped wedge cluster randomised designs [51].

Finally, although the study data were collected from 2009–2010, more recent evaluations of the study facilities by the authorship team indicate that prevalence of smoking among patients, and clinician provision of nicotine dependence treatment (including both patient report and medical record audit) are unlikely to have changed in this time [34, 47, 54]. Given that this study is the first to examine the efficacy of a clinical practice change intervention in increasing nicotine dependence treatment to smokers within inpatient psychiatric facilities, it provides a strong basis for future studies further develop the intervention design and outcome measures, and to explore potential differential effects of the intervention among specific subgroups of the patient population, including those with voluntary/involuntary admission status, and those who may be using other pharmacotherapies, such as varenicline and bupropion.

## Conclusions

This study is the first to examine the effectiveness of a multi-modal clinical practice change intervention in increasing recorded nicotine dependence treatment in inpatient psychiatric facilities. The use of inpatient psychiatric facilities as a setting for supporting and encouraging smokers to change behaviour may make an important contribution to addressing the recognised need to reduce smoking rates for this population group. Our results provide evidence that improvement in the routine recording of nicotine dependence treatment is achievable on a whole-of hospital basis and are sustainable in the short to medium term. However additional strategies are required to ensure all smokers are systematically identified and offered nicotine dependence treatment.

## Additional file

Additional file 1: (DOCX 228 kb)

## Abbreviations

CI: Confidence interval
FTE: Full time equivalent
ICD: International Classification of Diseases
NRT: Nicotine replacement therapy
OR: Odds ratio
SD: Standard deviation
