# Supplementary material for: Effectiveness of a clinical practice change intervention in increasing the provision of nicotine dependence treatment in inpatient psychiatric facilities: an implementation trial
Source: BMC Psychiatry. 2017 Feb 7;17:56. doi: 10.1186/s12888-017-1220-7 (PMC5297214; doi:10.1186/s12888-017-1220-7)
Supplement: Additional file 1: — (DOCX 228 kb) [file 12888_2017_1220_MOESM1_ESM.docx]

Additional file 1: **Effectiveness of a clinical practice change intervention in increasing the provision of nicotine dependence treatment in inpatient psychiatric facilities: an implementation trial**

Contents

[New South Wales Health Guide for the Management of Nicotine Dependent Inpatients- flowchart 3](#_Toc469939473)

[Unadjusted results of the segmented logistic regression 5](#_Toc469939474)

[Primary results of the intervention stratified by facility (100-bed facility vs. 125-bed facility) 6](#_Toc469939475)

**List of Tables**

[**Table 1.** Unadjusted results of segmented logistic regression showing differences in odds of reporting the key nicotine dependence treatment items within, and between the pre- (*n* = 1054), during (*n* = 2043) and post (*n* = 1078) periods. 5](#_Toc469939487)

[**Table 2.** Adjusted results of segmented logistic regression for the 100-bed facility (~2000 discharges per annum), showing differences in odds of reporting the key nicotine dependence treatment items within, and between the pre- (*n* = 827), during (*n* = 1598) and post (*n* = 833) periods. 6](#_Toc469939488)

[**Table 3.** Adjusted results of segmented logistic regression for the 125-bed facility (~750 discharges per annum), showing differences in odds of reporting the key nicotine dependence treatment items within, and between the pre- (*n* = 227), during (*n* = 445) and post (*n* = 245) periods. 7](#_Toc469939489)

New South Wales Health Guide for the Management of Nicotine Dependent Inpatients- flowchart
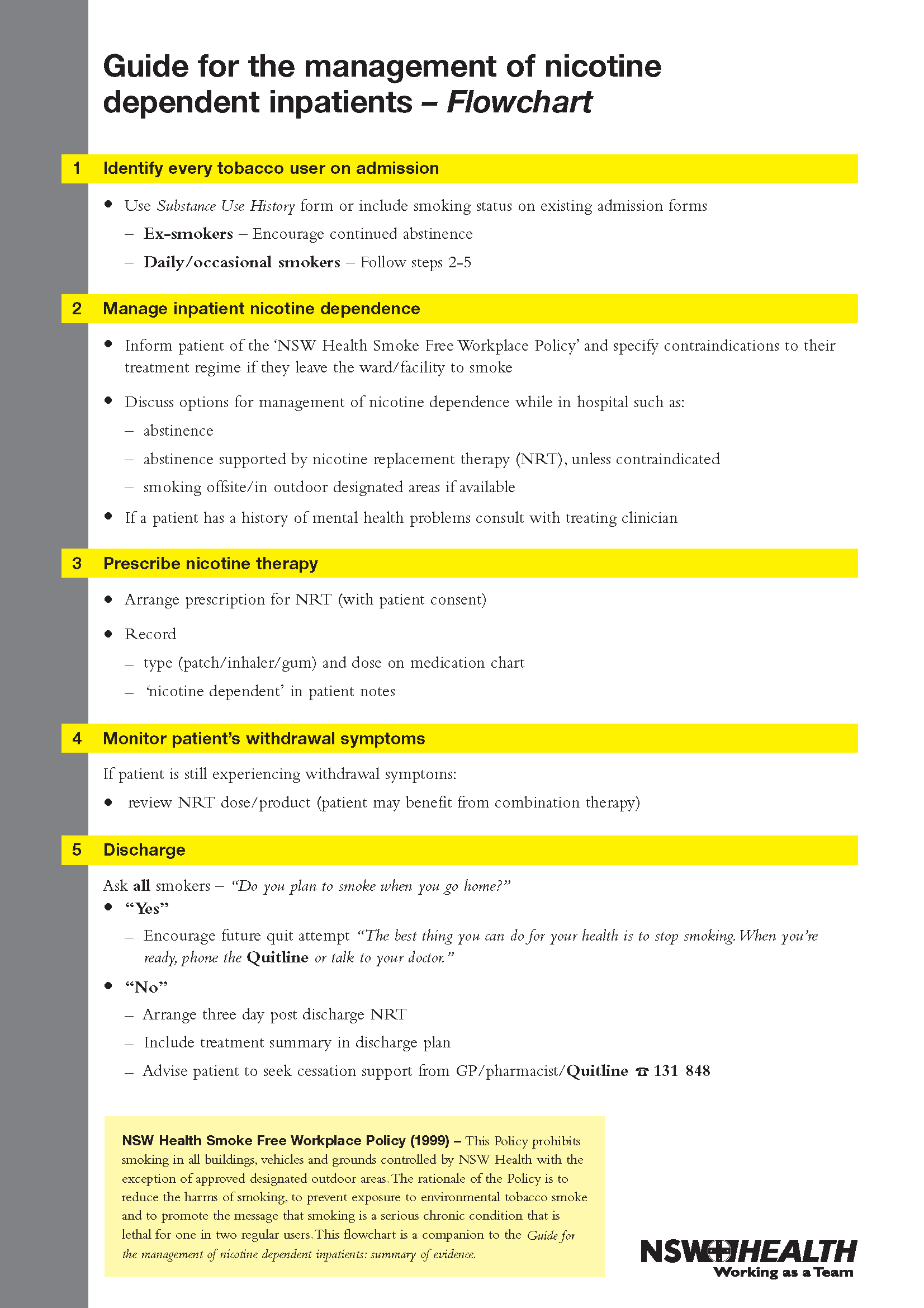


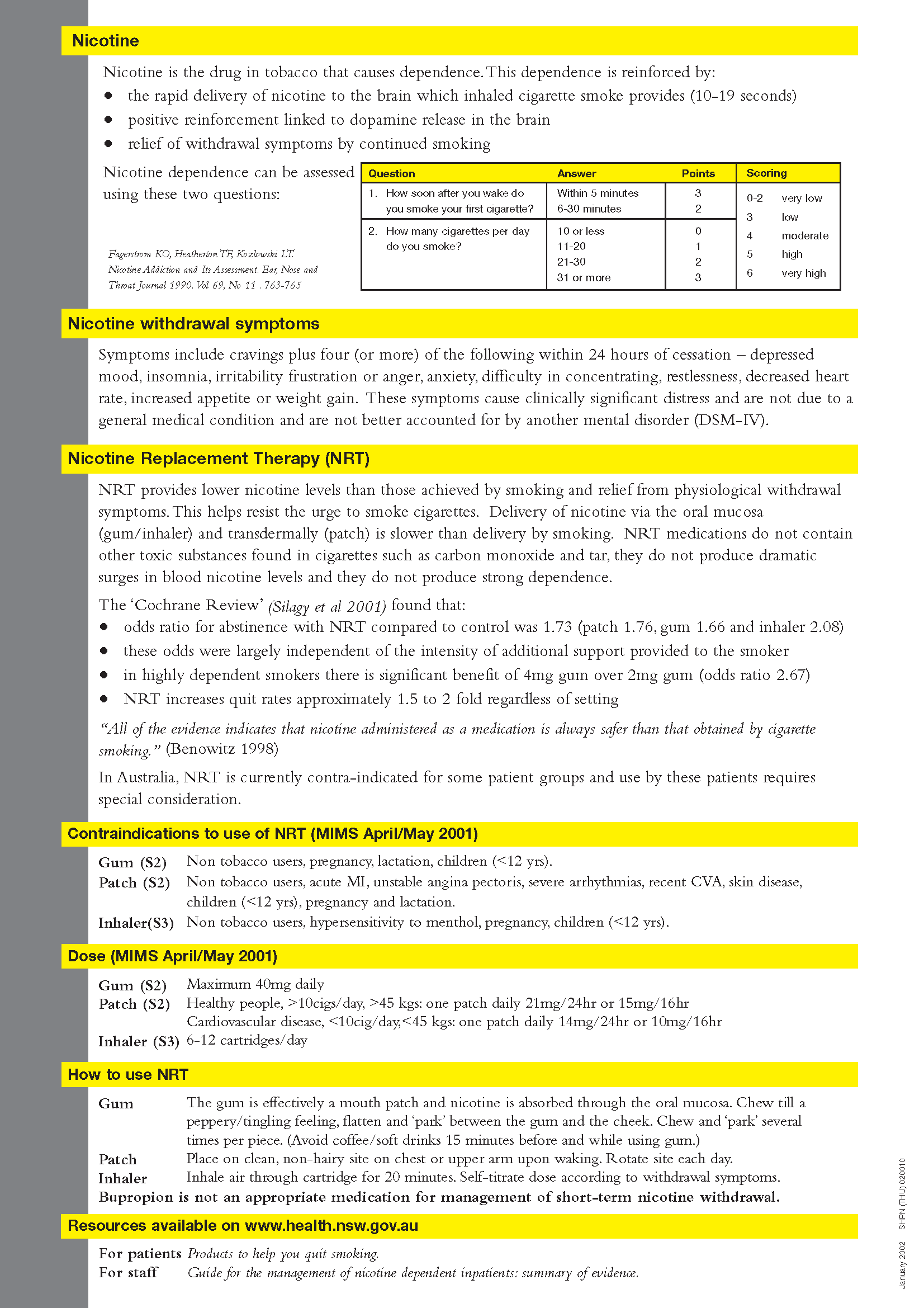


# Unadjusted results of the segmented logistic regression

Table 1. Unadjusted results of segmented logistic regression showing differences in odds of reporting the key nicotine dependence treatment items within, and between the pre- (*n* = 1054), during (*n* = 2043) and post (*n* = 1078) periods.

|  | **Slope OR^a^ within period (99%CI)** | | | **Difference in slope OR^a^ between periods (99%CI)** | | | |
| --- | --- | --- | --- | --- | --- | --- | --- |
| **Outcome** | **Pre intervention (*n*=1054)** | **Intervention period (*n*=2043)** | **Post intervention (*n*=1078)** | **Pre- intervention vs. intervention** | **Intervention vs. Post-intervention** | **Pre-intervention- vs. Post-intervention** | **End of pre-intervention vs. end of post-intervention** |
| Smoking status assessed | 0.99 (0.94, 1.05) | 1.02 (0.98, 1.07) | 1.02 (0.85, 1.24) | **1.03 (1.01, 1.05**)^**^ | 1.00 (0.83, 1.32) | 1.03 (0.81, 1.32) | **2.83 (1.47 to 5.44)**^**^ |
| Nicotine dependence assessed | 1.07 (0.95, 1.21) | **1.25 (1.12, 1.38)^**^** | **2.24 (1.65, 3.05)^**^** | **1.17 (1.10, 1.24)**^**^ | **1.80 (1.34, 2.42)**^**^ | **2.10 (1.42, 3.08)**^**^ | **109.67 (35.35, 340.22)**^**^ |
| Provided brief advice to quit | 1.08 (0.81, 1.43) | **1.33 (1.06, 1.66)^**^** | **2.29 (1.55, 3.39)^**^** | **1.23 (1.15, 1.32)**^**^ | **1.73 (1.24, 2.41)**^**^ | **2.13 (1.39, 3.27)**^**^ | **97.43 (30.99, 306.30)**^**^ |
| Prescribed NRT | 1.06 (0.96, 1.17) | **1.11 (1.03, 1.20)^**^** | **1.33 (1.06, 1.67)^**^** | **1.05 (1.02, 1.08)**^**^ | 1.19 (0.96, 1.48) | 1.25 (0.94, 1.66) | **21.29 (8.95, 50.62)**^**^ |
| Treatment for smoking on discharge | 1.05 (0.96, 1.15) | **1.09 (1.01, 1.18)**^**^ | 1.07 (0.83, 1.39) | **1.04 (1.00, 1.08)**^**^ | 0.98 (0.76, 1.27) | 1.03 (0.74, 1.43) | **13.54 (6.69, 27.40)^*^**^*^ |

^a^Figures presented here are unadjusted odds ratios.

^**^Indicates significant at *p* < .01.

OR = Odds ratio

CI = Confidence interval

NRT = Nicotine replacement therapy

# Primary results of the intervention stratified by facility (100-bed facility vs. 125-bed facility)

Table 2. Adjusted results of segmented logistic regression for the 100-bed facility (~2000 discharges per annum), showing differences in odds of reporting the key nicotine dependence treatment items within, and between the pre- (*n* = 827), during (*n* = 1598) and post (*n* = 833) periods.

|  | **Slope AOR^a^ within period (99%CI)** | | | **Difference in slope AOR^a^ between periods (99%CI)** | | | |
| --- | --- | --- | --- | --- | --- | --- | --- |
| **Outcome** | **Pre intervention (*n*=827)** | **Intervention period (*n*=1598)** | **Post intervention (*n*=833)** | **Pre- intervention vs. intervention** | **Intervention vs. Post-intervention** | **Pre-intervention- vs. Post-intervention** | **End of pre-intervention vs. end of post-intervention** |
| Smoking status assessed | 0.99 (0.93, 1.05) | 1.03 (0.98, 1.08) | 1.10 (0.90, 1.34) | **1.04 (1.01, 1.06**)^**^ | 1.07 (0.88, 1.30) | 1.10 (0.85, 1.43) | **3.94 (1.95 to 7.96)**^**^ |
| Nicotine dependence assessed | 1.05 (0.92, 1.20) | **1.24 (1.11, 1.39)^**^** | **2.52 (1.82, 3.49)^**^** | **1.18 (1.10, 1.27)**^**^ | **2.03 (1.48, 2.78)**^**^ | **2.40 (1.60, 3.62)**^**^ | **178.26 (54.14, 586.97)**^**^ |
| Provided brief advice to quit | 1.07 (0.81, 1.43) | **1.31 (1.05, 1.64)^**^** | **2.38 (1.60, 3.52)^**^** | **1.22 (1.14, 1.31)**^**^ | **1.81 (1.29, 2.52)**^**^ | **2.21 (1.43, 3.41)**^**^ | **97.43 (30.99, 306.30)**^**^ |
| Prescribed NRT | 1.06 (0.96, 1.17) | **1.10 (1.02, 1.19)**^+^**^**^** | **1.48 (1.18, 1.84)^**^** | **1.04 (1.01, 1.07)**^**^ | **1.34 (1.08, 1.65)**^+^**^**^** | **1.39 (1.05, 1.83)**^+^**^**^** | **22.78 (9.78, 53.06)**^**^ |
| Treatment for smoking on discharge | 1.06 (0.96, 1.17) | **1.11 (1.02, 1.21)**^**^ | 1.05 (0.78, 1.41)^±^ | 1.04 (1.00, 1.09) | 1.07 (0.88, 1.30) ^±^ | 1.10 (0.85, 1.43) ^±^ | **3.94 (1.95, 7.96)^*^**^*^ |

^a^Figures presented here are adjusted odds ratios.

^**^Indicates significant at *p* < .01.

+Indicates a significant result that was non-significant in the main analysis

±Indicates a non-significant result that was significant in the main analysis

OR = Odds ratio

CI = Confidence interval

NRT = Nicotine replacement therapy

Table 3. Adjusted results of segmented logistic regression for the 125-bed facility (~750 discharges per annum), showing differences in odds of reporting the key nicotine dependence treatment items within, and between the pre- (*n* = 227), during (*n* = 445) and post (*n* = 245) periods.

|  | **Slope AOR^a^ within period (99%CI)** | | | **Difference in slope AOR^a^ between periods (99%CI)** | | | |
| --- | --- | --- | --- | --- | --- | --- | --- |
| **Outcome** | **Pre intervention (*n*=227)** | **Intervention period (*n*=445)** | **Post intervention (*n*=245)** | **Pre- intervention vs. intervention** | **Intervention vs. Post-intervention** | **Pre-intervention- vs. Post-intervention** | **End of pre-intervention vs. end of post-intervention** |
| Smoking status assessed | 1.00 (0.89, 1.12) | 0.99 (0.91, 1.09) | 0.97 (0.67, 1.40) | 0.99 (0.95, 1.04)^±^ | 0.98 (0.68, 1.40) | 0.97 (0.61, 1.55) | 0.83 (0.23, 2.97)^±^ |
| Nicotine dependence assessed | 1.28 (0.88, 1.85) | **1.40 (1.04, 1.89)^**^** | 1.23 (0.50, 3.04)^±^ | 1.09 (0.96, 1.24)^±^ | 0.88 (0.37, 2.09)^±^ | 0.96 (0.31, 2.98)^±^ | 12.67 (0.35, 456.95)^±^ |
| Provided brief advice to quit | 1.00 (0.00, - )^#^ | 1.53 (0.00, - )^#±^ | 0.57 (0.00, - )^#±^ | 1.53 (0.79, 2.99)^+±^ | 0.37 (0.06, 2.25)^+±^ | 0.57 (0.06, 5.51)^+±^ | 356e9 (285797, 444e15)^#±^ |
| Prescribed NRT | 1.13 (0.73, 1.74) | 1.25 (0.88, 1.76)^±^ | 0.45 (0.17, 1.19)^±^ | 1.11 (0.98, 1.26)^±^ | 0.36 (0.14, 0.90)^±^ | 0.40 (0.12, 1.32)^±^ | 0.70 (0.10, 39.55)^±^ |
| Treatment for smoking on discharge | 1.00 (0.81, 1.23) | 1.02 (0.86, 1.22) | 1.18 (0.67, 2.06)^±^ | 1.03 (0.95, 1.11) | 1.15 (0.67, 1.97)^±^ | 1.18 (0.58, 2.39)^±^ | 3.60 (0.49, 26.26)^±^ |

^a^Figures presented here are adjusted odds ratios.

^**^Indicates significant at *p* < .01.

±Indicates a non-significant result that was significant in the main analysis

-Indicates a result was not estimable due to insufficient numbers

#Indicates results should be interpreted with caution as there were insufficient cell counts for the segmented logistic regression analysis

OR = Odds ratio

CI = Confidence interval

NRT = Nicotine replacement therapy
